# AI‐driven preoperative risk assessment in kidney cancer surgery: A comparative feasibility study of machine learning models

**DOI:** 10.1002/bco2.70080

**Published:** 2025-09-25

**Authors:** Julia Mühlbauer, Luise Gottstein, Luisa Egen, Caelan Haney, Alexander Studier‐Fischer, Evangelia Christodoulou, Giovanni E. Cacciamani, Keno März, Lena Maier‐Hein, Stephan Maurice Michel, Allison Quan, Karl‐Friedrich Kowalewski

**Affiliations:** ^1^ Department of Urology and Urosurgery, University Medical Center Mannheim Medical Faculty Mannheim at Heidelberg University Germany; ^2^ Division of Intelligent Systems and Robotics in Urology (ISRU) German Cancer Research Center (DKFZ) Heidelberg Heidelberg Germany; ^3^ DKFZ Hector Cancer Institute at the University Medical Center Mannheim Mannheim Germany; ^4^ German Cancer Research Center (DKFZ) Heidelberg, Division of Intelligent Medical Systems Heidelberg Germany; ^5^ USC Institute of Urology, Catherine & Joseph Aresty Department of Urology, Keck School of Medecine University of Southern California (USC) Los Angeles CA USA; ^6^ HIDSS4Health – Helmholtz Information and Data Science School for Health, Karlsruhe Heidelberg Germany; ^7^ National Center for Tumor Diseases (NCT) Heidelberg a partnership between DKFZ and Heidelberg University Hospital Heidelberg Germany; ^8^ Faculty of Mathematics and Computer Science Heidelberg University Heidelberg Germany; ^9^ Department of Urology Queen's University Kingston Canada

**Keywords:** Complications, Kidney cancer, Kidney function, Machine Learning, Nephrectomy, Prognosis model, Renal cell carcinoma

## Abstract

**Background and Objective:**

Preoperative risk stratification in renal tumour surgery is essential to enable risk‐adjusted postoperative patient monitoring. Machine learning (ML) models predicting major complications (MCs) and acute kidney injuries (AKIs) following partial (PN) or radical nephrectomy (RN) have not been made, nor have they been compared with traditional logistic regression models.

**Design, setting and participants:**

A total of 963 patients who underwent PN and RN between January 2017 and March 2023 at the University Medical Center Mannheim were included. The dataset consisted of 30 variables of interest– 18 descriptive and 12 predictor variables, which allowed for 7 predictor variables per event. The dataset was pre‐processed, and ML models were created for MC and AKI. The selected models included Random Forest (RF), Support Vector Machines (SVMs), Stochastic Gradient Boosting, Neural Networks (NNs) and Elastic Net Logistic Regression models (ENETs).

**Results and limitations:**

For major complications, the NN model had the best model fitting, with an AUROC of 0.762 [95%CI 0.611–0.912], a sensitivity of 0.86 [95%CI 0.80–0.92] and a Brier score of 0.17 [95%CI 0.11–0.23]. For AKI, the best fit model was created using a NN with an AUROC of 0.717 [95%CI 0.611–0.823], a sensitivity of 0.82 [95%CI 0.74–0.90] and a Brier score of 0.24 [95%CI 0.17–0.31]. The best performing models for both outcomes outperformed the ENETs.

**Conclusions:**

The ML models provide valuable information for preoperative risk stratification of patients undergoing renal tumour surgery. This study suggests that NNs are the most appropriate models to stratify patients regarding the occurrence of MCs and AKIs, respectively. The models are made publicly available for reproducibility.

## 1 INTRODUCTION

In renal cell carcinoma (RCC), resection of the tumour or– if organ preservation is not technically feasible– the removal of the tumour‐bearing kidney represents the current therapeutic gold standard.^1^ Despite the high standardization of surgical approaches, renal tumour surgery (RTS) remains a challenging procedure with a considerable risk of adverse events.^1^ This applies to both organ‐preserving partial nephrectomy (PN) as well as radical nephrectomy (RN).^1^ Apart from immediate perioperative major complications (MCs), this also includes acute kidney injuries (AKIs), occurring in up to 35% postoperatively.^1^ In this context, an accurate preoperative risk stratification is required to optimize the individual perioperative management and monitoring, but reliable tools remain an unmet need.

Various studies have applied univariate and multivariate logistic regression models to investigate and identify predictors of post‐operative MCs and AKIs, so that they can serve as prediction models.^2^, ^3^, ^4^ However, they have not been implemented into clinical practice for a variety of reasons and are subject to considerable limitations, such as small sample sizes, out‐of‐date datasets and the lack of external validation.^2^, ^3^, ^4^ Modern computational methods, such as artificial intelligence (AI) and machine learning (ML), have the potential to overcome these limitations, create optimized prediction models and bear potential to improve patient outcomes.^5^, ^6^ Compared to traditional methods, ML can detect associations that do not appear to be obvious to the subjective investigator.^5^, ^6^ This also includes, but is not limited to, the prediction of surgical complications in general, acute renal failure after cardiac surgery and long‐term consequences of diseases, such as diabetic retinopathy.^7^ Related to RTS, a recent study was able to show that the application of ML models to predict recurrence following surgical resection of RCC resulted in better prediction than that of current validated models available in clinical practice.^7^ Regarding the prediction of surgical complexity, a small pilot study failed to show superiority of ML models focusing on different combinations of preoperative medical imaging features compared to a clinical benchmark model for predicting postoperative complications (Clavien‐Dindo‐Classification [CDC] ≥ 2) after RTS.^7^ However, the main area of research involving ML in RCC concerns the differentiation between benign and malignant renal tumours, nuclear grade prediction and gene expression‐based molecular signatures.^8^, ^9^ To our knowledge, there is currently no large‐scale study that focused on the establishment of ML models predicting MCs and AKIs following RTS, nor compared them to traditional logistic regression models. Therefore, this study aims to address the need for an accurate pre‐operative risk stratification method, to improve management and identify long‐term strategies for patient care and decision‐making.

## 2 PATIENTS AND METHODS

### 2.1 Study design and patient selection

**FIGURE 1:**
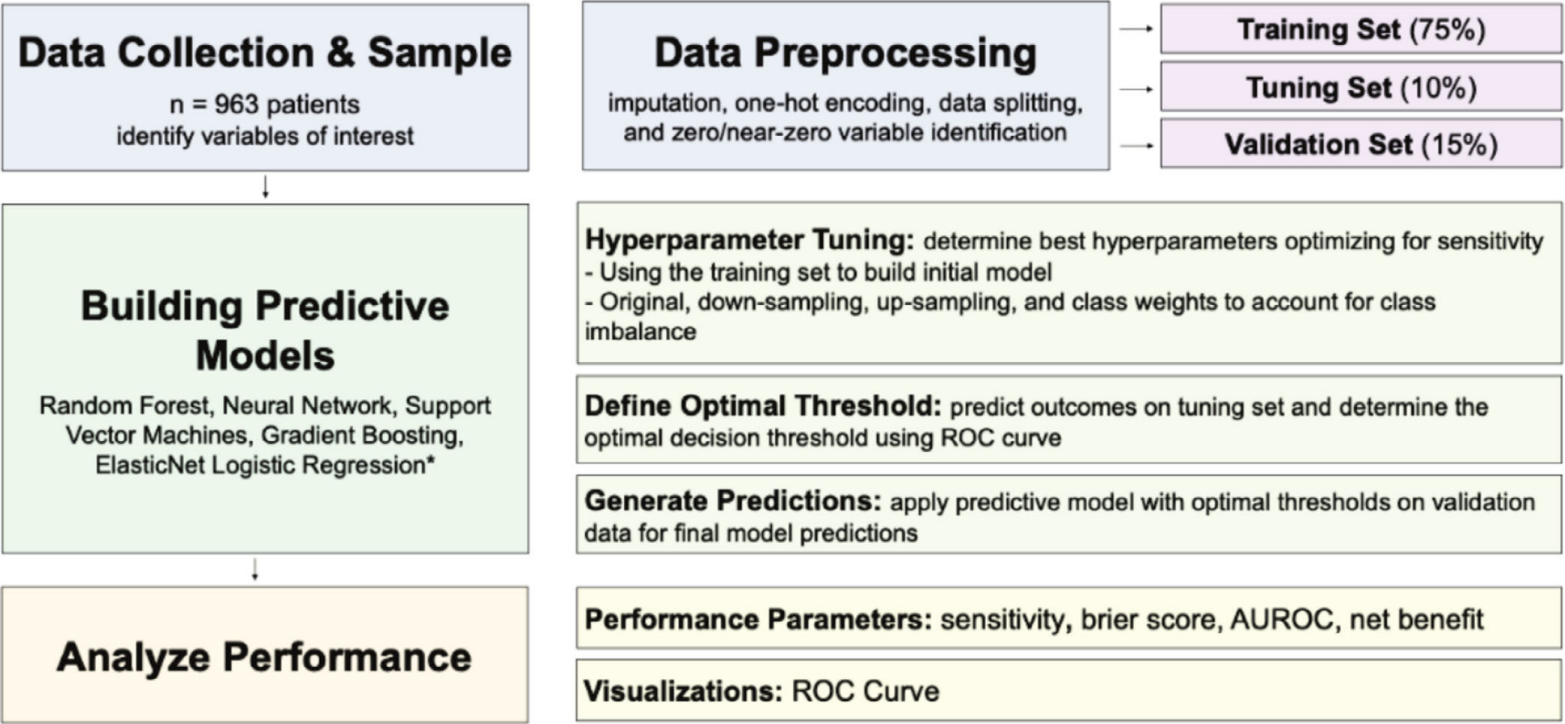
Flow chart of the methods: data collection, model development and performance analysis.

After institutional ethical approval, all patients who underwent PN or RN between January 2017 and March 2023 were retrospectively extracted from a comprehensive database of all patients undergoing surgery for renal tumours maintained by our tertiary care centre. There were no specific exclusion criteria resulting in a study cohort of n = 963 patients. A flow chart of the methods used including data collection, model development and performance analysis is shown in Figure 1. The dataset consisted of 30 variables of interest ‐ 18 descriptive and 12 predictor variables, which allowed for 7 predictor variables per event (Table A1).^10^ The predicted study endpoints were MCs (86 events, 8.9%) and AKIs (322 events, 33.4%). Data pre‐processing and processing were conducted in R v4.3.1 (2023) using the caret package, and methods followed the guidelines for Transparent Reporting of a multivariable prediction model for Individual Prognosis Or Diagnosis with AI (TRIPOD+AI).^11^ The TRIPOD‐AI checklist of this study is listed in Table A2.

### 2.2 Study endpoints

Perioperative complications were defined by the Clavien‐Dindo Classification (CDC),^12^ and complications of grade ≥ 3 were classified as MCs. All complications occurring between the surgery and postoperative discharge from the hospital were considered.

Renal function (RF) was assessed by the preoperative and postoperative estimated glomerular filtration (eGFR) rates as well as by the creatinine serum levels. Based on these values, the postoperative AKI stage according to the Acute Kidney Injury Criteria Network (AKIN)‐criteria was calculated.^13^

### 2.3 Data Preprocessing

Preprocessing included k‐nearest neighbour imputation, one‐hot encoding, data splitting and zero/near‐zero variable identification, and was conducted to format raw data for ML interpretation.^11^ Data splitting separated the dataset into a training set (75%) and testing set that was further subdivided into a validation set (10%) and testing set (15%).^11^

The training set was the data that was used to cross‐validate and adjust the tuning parameters of the data to the ML model. In addition to testing regular model testing, to handle the class imbalance, various sampling methods were applied to the data fitting including up‐sampling, down‐sampling and class‐weighting based on the imbalance ratio (calculated from class imbalance). The test set was used to make preliminary predictions using the trained models. Based on preliminary results, the optimal threshold was determined and applied to the model. When the preliminary results are satisfactory, the model was exposed to the validation set for final predictions. This allows the validation set to be reserved and only seen by the model for final predictions.

### 2.4 Machine Learning Models

Random Forest (RF), Neural Network (NN), Support Vector Machines (SVM), Gradient Boosting (GBM) and Elastic Net Regression (ENET) ML models showed the greatest predictive potential in similar literature and thus were created for both major complications and AKI.^14^, ^15^ The models' unique tuning parameters (Table A3), sampling methods, weighting and decision metrics were optimized for each model to improve their prediction accuracy, indicated by the performance parameters. The models were created to optimize for sensitivity to minimize the occurrence of predicting false negative results. The rationale here is that missing an event is worse than overestimating events (or from a clinical perspective: missing one complication is worse than monitoring one additional patient without complications).

### 2.5 Performance Parameters

The models were assessed based on sensitivity as the primary performance parameters. Other performance parameters that were considered include Brier score, net benefit and the area under the receiver operating curve (AUROC), all with the accompanying 95% confidence intervals [CI].^10^, ^11^, ^16^ Brier scores measure the calibration of a model and range from 0 to 1, with 0 indicating the greatest accuracy of prediction.^17^ Net benefit measures the usefulness of applying the ML model for decision, with higher values indicating the model provides greater benefit than harm. These variables were output in a confusion matrix and assess the discriminatory power (whether the event occurs) and calibration (agreement between frequency of observed events and predicted probabilities) of the prediction model. Only the best‐calibrated version of each model was kept for comparison and discussion.

The models' strengths, weaknesses and best ML model were determined through comparison of performance metrics.^10^, ^11^

## 3 RESULTS

### 3.1 Cohort characteristics

The present study included 963 patients who underwent RTS between January 2017 and March 2023. Table A4 summarizes all the patient characteristics collected and included in the study. The listed variables were evaluated as potentially relevant predictors for the ML models, except for MC and AKI as the outcome parameters.

### 3.2 Major complications

**FIGURE 2:**
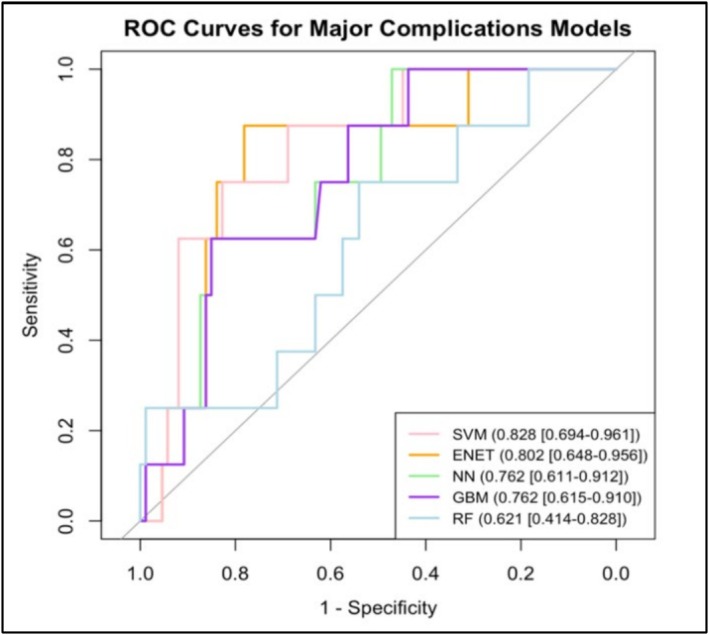
Superimposed ROC curves of the best models predicting major complications optimized for sensitivity. Bottom‐right legend coloured by model and noting AUROC [95% CI].

The final predictions and results were determined from applying the best models and threshold values on the unseen validation set. Figure 2 and Table 1 outline the performance parameters calculated for the best‐performing models created for predicting major complications. The NN model had the highest sensitivity of 0.86 [95%CI 0.80–0.92] indicating the best overall model fit optimizing for sensitivity. The NN model for major complications had an acceptable AUROC of 0.762 [95%CI 0.611–0.912] and a strong Brier score of 0.17 [95%CI 0.11–0.23]. The net benefit value indicates that NN provides the greatest benefit compared to the other models. The model that output the best AUROC for major complications was SVM, with an AUROC of 0.828 [95%CI 0.694–0.961], a sensitivity value of 0.83 [95%CI 0.76–0.90] and a Brier score of 0.18 [95%CI 0.12–0.24]. The net benefit of this model was 0.08 [95%CI ‐0.05‐0.30].

**TABLE 1:** Sensitivity, Brier score, AUROC and net benefit values [95% CI] for best performing models predicting major complications, optimized for sensitivity.

Model	Sensitivity [95%CI]	Brier Score [95%CI]	AUROC [95%CI]	Net Benefit [95%CI]
**Random Forest**	0.53 [0.44–0.62]	0.48 [0.40–0.57]	0.621 [0.414–0.828]	0.05 [−0.04–0.14]
**Support Vector Machines**	0.83 [0.76–0.90]	0.18 [0.12–0.24]	0.828 [0.694–0.961]	0.08 [−0.05–0.30]
**Neural Network**	0.86 [0.80–0.92]	0.17 [0.11–0.23]	0.762 [0.611–0.912]	0.14 [−0.02–0.37]
**Stochastic Gradient Boosting**	0.83 [0.75–0.89]	0.19 [0.12–0.26]	0.762 [0.615–0.910]	0.07 [−0.05–0.25]
**Elastic Net Regression**	0.80 [0.73–0.87]	0.21 [0.15–0.28]	0.802 [0.648–0.956]	0.05 [−0.11–0.12]

Following the construction of the machine learning models, an ENET was built for both outcomes to allow for the comparison of the ML models to classic logistic regression models. Outlined in Figure 2 and Table 1, the ENET model for major complications had a sensitivity of 0.80 [95%CI 0.73–0.87] and AUROC of 0.802 [95%CI 0.648–0.956], making it the second‐best in overall fit, but only outperforming the RF model in terms of sensitivity. Similarly, the model had a Brier score of 0.21 [95%CI 0.15–0.28] and low net benefit of 0.05 [95%CI ‐0.11‐0.12], which only provided improvement from the RF model.

**FIGURE A1:**
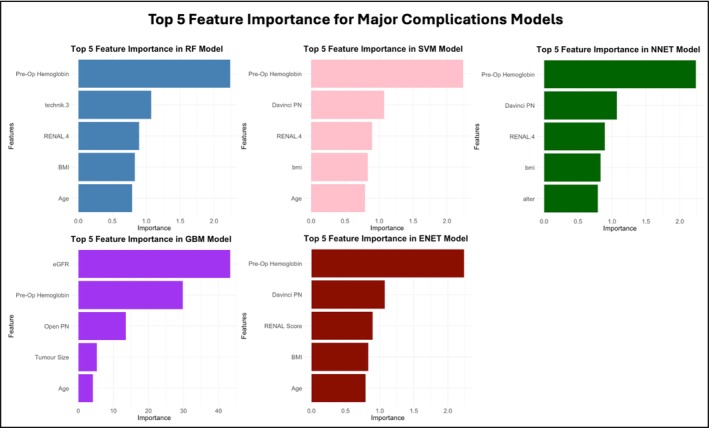
The importance of Variables in each prediction model for major complications. (A) Random Forest (RF). (B) Support Vector Machines (SVM). (C) Neural Network. (D) Stochastic Gradient Boosting (GBM). (E) Elastic Net Regression.

Figure A1 visualizes the relative influence of the top five most important features in each of the models for major complications. Open PN technique, eGFR and pre‐operative haemoglobin are among the most important across all models.

### 3.3 Acute kidney injury

**FIGURE 3:**
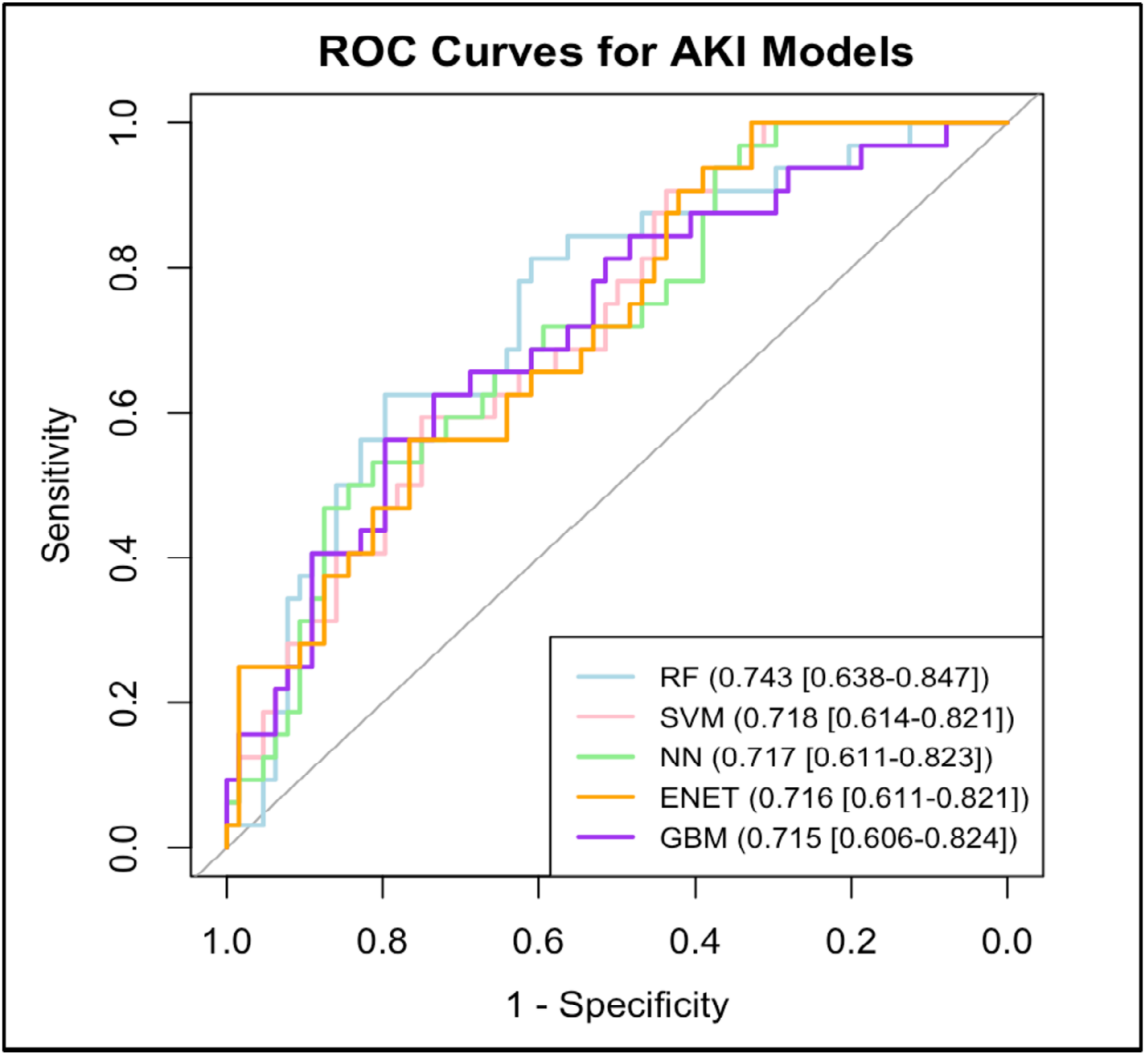
Overlapping ROC curves of the best models predicting AKI optimized for sensitivity. Bottom‐right legend coloured by model and noting AUROC [95% CI].

Figure 3 and Table 2 outline the performance parameters calculated for the best‐performing models created for predicting AKI. The NN model provided the best overall fit for the predictive model for AKI, as signified by the AUROC of 0.717 [95% CI 0.611–0.823] and sensitivity of 0.82 [95% CI 0.74–0.90]. The NN model also provided a moderate Brier score of 0.24 [95% CI 0.17–0.31] and strong net benefit of 0.24 [95% CI 0.04–0.42]. The model that output the greatest AUROC for predicting AKI was RF with an AUROC of 0.743 [95% CI 0.638–0.847] sensitivity value of 0.76 [95% CI 0.67–0.85], moderate Brier score of 0.32 [95% CI 0.24–0.40] and moderate net benefit of 0.24 [95% CI 0.06–0.42]. All the other machine learning algorithms show no significant difference between one another, with the AUROCs all being between 0.715 and 0.718 with overlapping confidence intervals.

**TABLE 2:** Sensitivity, Brier score, AUROC and net benefit values [95% CI] for best performing models predicting AKI, optimized for sensitivity.

Model	Sensitivity	Brier Score	AUROC	Net Benefit
**Random Forest**	0.76 [0.67–0.85]w	0.32 [0.24–0.40]	0.743 [0.638–0.847]	0.24 [0.06–0.42]
**Support Vector Machines**	0.70 [0.61–0.80]	0.35 [0.28–0.43]	0.718 [0.614–0.821]	0.15 [−0.01–0.31]
**Neural Network**	0.82 [0.74–0.90]	0.24 [0.17–0.31]	0.717 [0.611–0.823]	0.24 [0.04–0.42]
**Stochastic Gradient Boosting**	0.72 [0.63–0.81]	0.38 [0.29–0.45]	0.715 [0.606–0.824]	0.27 [0.10–0.42]
**Elastic Net Regression**	0.67 [0.57–0.76]	0.41 [0.33–0.49]	0.716 [0.611–0.821]	0.21 [0.05–0.36]

Described in Figure 3 and Table 2, the ENET model for AKI had a sensitivity of 0.67 [95% CI 0.57–0.76] and an AUROC of 0.716 [0.611–0.821]. This model provided the worst sensitivity of all predictive models for AKI, while the AUROC was on par with the other models. The model provided a weak Brier score of 0.41 [95% CI 0.33–0.49] and a moderate net benefit of 0.21 [95% CI 0.05–0.36]. The weak Brier score signifies very low model calibration.

**FIGURE A2:**
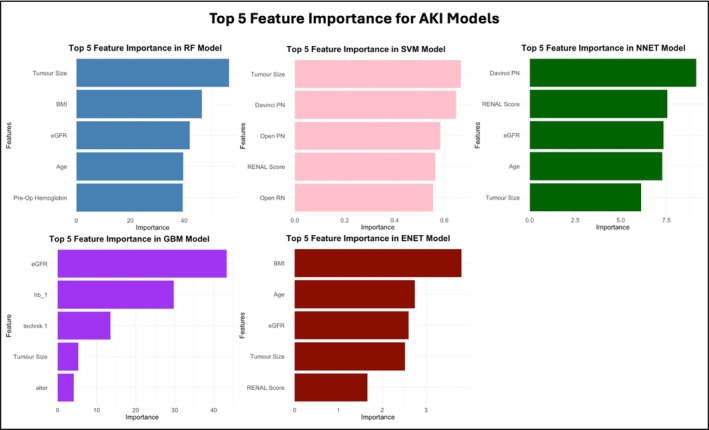
The importance of variables in each prediction model for AKI. (A) Random Forest (RF). (B) Support Vector Machines (SVM). (C) Neural Network. (D) Stochastic Gradient Boosting (GBM). (E) Elastic Net Regression.

Figure A2 visualizes the relative influence of the top five most important features in each of the models for AKI. Tumour size, BMI and utilization of robot‐assisted PN are among the most important across all models.

## 4 DISCUSSION

In RTS, preoperative risk‐stratification of patients is required to identify patients at high risk for serious postoperative complications and unfavourable functional outcomes in terms of AKIs to allow personalized, risk‐adjusted perioperative management and monitoring. In the era of AI, this study represents an initial large‐scale investigation to establish ML models predicting MCs and AKIs following RTS and comparing them to traditional logistic regression models. We found that NNs and RF are the most appropriate models to stratify patients regarding the occurrence of MCs and AKIs, respectively. Additionally, for MCs, the most important variables were open PN technique, pre‐operative eGFR and pre‐operative haemoglobin. For AKI, the most important variables were tumour size, BMI and utilization of robot‐assisted PN. This information will help guide future clinical applications of these models.

With the increasing digitalization of medical data, the use of ML offers the opportunity to identify correlations that are not apparent to the subjective examiner, thus providing an objective basis for decision‐making and risk stratification.^17^

Preliminary model performance parameters indicate that NN provides strong model fitting for the prediction of major complications (AUROC 0.762 [95% CI 0.611–0.912]) and a high sensitivity of 0.86 [95% CI 0.80–0.92]. This indicates a strong discriminatory ability of the NN model to identify patients with major complications and those without. This is supported by the high sensitivity, highlighting its effectiveness in correctly identifying patients at risk of major complications. The NN model's Brier score of 0.17 [95% CI 0.11–0.23] indicates that the model shows strong accuracy. Nevertheless, major complications were only present in 8.9% of patients in the dataset, indicating extreme class imbalance. Despite being the highest compared to the other models, the objectively low net benefit of the NN model, 0.14 [95% CI ‐0.02‐0.37], may indicate that the model has been overfit and is showing inflated performance due to ease of predicting against the outcome.^18^ Furthermore, it cannot be said in confidence that the ML model outperformed the logistic model, as the ENET model performed to a similar standard (AUROC 0.802 [95% CI 0.648–0.956], sensitivity 0.80 [95%CI 0.73–0.87]. The Brier score of the ENET model, 0.21 [95% CI 0.15–0.28], was comparable to that of the NN model; however, the net benefit of the ENET model, 0.05 [95%CI ‐0.11‐0.12], was lower than NN. This discrepancy in net benefit values may be due to imbalance affecting the ENET model to a slightly greater extent than the NN model or a difference in the nature of the models' hyperparameters' ability to manage class imbalance in decision‐making.^15^, ^19^ However, the determination of whether a benefit exists in using a logistic regression model compared to an ML model in predicting major complications requires further elucidation. At this point, both models will continue to be developed, compared and assessed for future clinical applications.

Preliminary model performance parameters for the prediction of AKI indicate NN to have strong model fitting (AUROC 0.717 [95% CI 0.611–0.823]) and high sensitivity of 0.82 [95% CI 0.74–0.90]. Therefore, there is a strong ability of the NN model to discriminate between the positive and negative outcomes of AKI with a high degree of true positive predictions. The brier score of the 0.24 [95%CI 0.17–0.31], while objectively not very strong, is the best of all AKI models and is still acceptable based on other published literary values.^4^, ^14^, ^15^ Additionally, the net benefit of 0.24 [95% CI 0.04–0.42] shows a robust margin of benefit.^20^ The ENET logistic regression model for AKI, however, was greatly outperformed by NN and the other ML models (AUROC 0.716 [95% CI 0.611–0.821], sensitivity 0.67 [95% CI 0.57–0.76]). Furthermore, the ENET model demonstrated the highest Brier score of 0.41 [95% CI 0.33–0.49] and relatively low net benefit of 0.21 [95% CI 0.05–0.36]. This suggests that for AKI, ML models may have greater predictive power worth considering over traditional logistic regression models.^15^

The models created for the prediction of major complications generally demonstrate greater sensitivity, but weaker AUROC, Brier scores and net benefits compared to models created for AKI. This may be attributed to AKI being present in 33.4% of the patients in the dataset, creating significantly less class imbalance than the 8.9% of major complications. The models created for major complications may be experiencing a greater degree of overfitting and bias towards the majority class.^18^

However, there are limitations that must be considered. First, our cohort size of about 1000 patients, which seems rather small in comparison to other multicentric investigations, may have reduced the ability of the ML models to learn complex relations from the data. Thus, our study requires external validation on larger datasets to ensure the reproducibility of our results. Second, the retrospective data collection, which may have influenced the data quality and completeness, must be considered. Furthermore, the lack of transparency in ML algorithms in general makes it difficult to comprehend the cause‐effect relationship for the investigator and the patient. This could be one reason why the application of ML models in clinical practice is difficult and thus not yet widely used. A future goal should be to translate the algorithms into a form that can be used easily and understandably by everyone in the future. Finally, the not yet widely established electronic, uniform data collection of patients in most countries also represents a hurdle in being able to use ML algorithms easily and quickly in everyday clinical practice.

## 5 CONCLUSION

Identifying surgical patients at a high risk of postoperative unfavourable outcomes and providing personalized precision medicine‐based monitoring and management strategies provides a pathway for reducing patient morbidity and mortality. The results of this study align with studies in other surgical fields. However, it presents novel findings in the field of ML and urology regarding accurate pre‐operative risk stratification as it is the first of its kind to predict MCs and AKI following kidney cancer surgery. The models will be made available for preoperative planning and risk assessment. Thus, future directions include external validation and creating a nomogram calculator for clinical application of the MCs prediction model.

## AUTHOR CONTRIBUTIONS

Julia Mühlbauer: protocol/project development, data analysis and interpretation, manuscript writing/editing.

Luise Gottstein: data collection.

Luisa Egen: data collection, critical scientific input, revision and editing.

Caelan Haney: critical scientific input, revision and editing.

Alexander Studier‐Fischer: critical scientific input, revision and editing.

Evangelia Christodoulou: critical scientific input, revision and editing.

Keno März: critical scientific input, revision and editing.

Lena Maier‐Hein: critical scientific input, revision and editing.

Stephan Maurice Michel: critical scientific input, revision and editing.

Allison Quan: data analysis and interpretation, manuscript writing/editing.

Karl‐Friedrich Kowalewski: protocol/project development, data analysis and interpretation, revision and editing, supervision and mentorship.

## CONFLICT OF INTEREST STATEMENT

All authors declare no conflict of interest.

## FUNDING SOURCES

The authors received no financial support for the research, authorship and publication of this article.

## Data Availability

All data generated or analysed during this study are included in this article and its supplementary files. Further enquiries can be directed to the corresponding author.

## Appendix

**TABLE A1:** Study variables grouped as outcome variables, predictor variables and descriptive variables.

Study Variables		
Outcome Variables	Predictor Variables	Descriptive Variables
Major complications (CDC)	Age	Pre‐operative creatinine
Acute kidney injury (AKI score)	Sex	Pre‐operative eGFR
	BMI	Pre‐operative CKD stage
	Pre‐operative haemoglobin	PADUA
	Solitary kidney (yes/no)	pT stage
	ASA classification	pN stage
	MAP score	Tumour grade
	Multifocality of tumour	R status
	Tumour size	Histological subtype
	RENAL score	Surgery duration
	Surgical approach/technique	Estimated blood loss
	Surgeon experience	Ischemia
		Ischemia time
		Drainage
		Intraoperative pleural opening
		Intraoperative opening of collective duct system
		Use of tissue adhesive sealant
		Haemoglobin drop
		Duration of hospital stay

**TABLE A2:** TRIPOD‐AI checklist^**11**^.

Section/topic	Item	Development/evaluation*	Checklist item
Title			
**Title**	1	D;E	Identify the study as developing or evaluating the performance of a multivariable prediction model, the target population and the outcome to be predicted
**Abstract**			
**Abstract**	2	D;E	See TRIPOD+AI for Abstracts checklist
**Introduction**			
**Background**	3a	D;E	Explain the healthcare context (including whether diagnostic or prognostic) and rationale for developing or evaluating the prediction model, including references to existing models
	3b	D;E	Describe the target population and the intended purpose of the prediction model in the context of the care pathway, including its intended users (eg, healthcare professionals, patients, public)
	3c	D;E	Describe any known health inequalities between sociodemographic groups
**Objectives**	4	D;E	Specify the study objectives, including whether the study describes the development or validation of a prediction model (or both)
**Methods**			
**Data**	5a	D;E	Describe the sources of data separately for the development and evaluation datasets (eg, randomized trial, cohort, routine care or registry data), the rationale for using these data and representativeness of the data
	5b	D;E	Specify the dates of the collected participant data, including start and end of participant accrual; and, if applicable, end of follow‐up
**Participants**	6a	D;E	Specify key elements of the study setting (eg, primary care, secondary care, general population) including the number and location of centres
	6b	D;E	Describe the eligibility criteria for study participants
	6c	D;E	Give details of any treatments received, and how they were handled during model development or evaluation, if relevant
**Data preparation**	7	D;E	Describe any data pre‐processing and quality checking, including whether this was similar across relevant sociodemographic groups
**Outcome**	8a	D;E	Clearly define the outcome that is being predicted and the time horizon, including how and when assessed, the rationale for choosing this outcome, and whether the method of outcome assessment is consistent across sociodemographic groups
	8b	D;E	If outcome assessment requires subjective interpretation, describe the qualifications and demographic characteristics of the outcome assessors
	8c	D;E	Report any actions to blind assessment of the outcome to be predicted
**Predictors**	9a	D	Describe the choice of initial predictors (eg, literature, previous models, all available predictors) and any pre‐selection of predictors before model building
	9b	D;E	Clearly define all predictors, including how and when they were measured (and any actions to blind assessment of predictors for the outcome and other predictors)
	9c	D;E	If predictor measurement requires subjective interpretation, describe the qualifications and demographic characteristics of the predictor assessors
**Sample size**	10	D;E	Explain how the study size was arrived at (separately for development and evaluation), and justify that the study size was sufficient to answer the research question. Include details of any sample size calculation
**Missing data**	11	D;E	Describe how missing data were handled. Provide reasons for omitting any data
**Analytical methods**	12a	D	Describe how the data were used (eg, for development and evaluation of model performance) in the analysis, including whether the data were partitioned, considering any sample size requirements
	12b	D	Depending on the type of model, describe how predictors were handled in the analyses (functional form, rescaling, transformation or any standardization)
	12c	D	Specify the type of model, rationale†, all model‐building steps, including any hyperparameter tuning, and method for internal validation
	12d	D;E	Describe if and how any heterogeneity in estimates of model parameter values and model performance was handled and quantified across clusters (eg, hospitals, countries). See TRIPOD‐Cluster for additional considerations‡
	12e	D;E	Specify all measures and plots used (and their rationale) to evaluate model performance (eg, discrimination, calibration, clinical utility) and, if relevant, to compare multiple models
	12f	E	Describe any model updating (eg, recalibration) arising from the model evaluation, either overall or for particular sociodemographic groups or settings
	12 g	E	For model evaluation, describe how the model predictions were calculated (eg, formula, code, object, application programming interface)
**Class imbalance**	13	D;E	If class imbalance methods were used, state why and how this was done, and any subsequent methods to recalibrate the model or the model predictions
**Fairness**	14	D;E	Describe any approaches that were used to address model fairness and their rationale
**Model output**	15	D	Specify the output of the prediction model (eg, probabilities, classification). Provide details and rationale for any classification and how the thresholds were identified
**Training versus evaluation**	16	D;E	Identify any differences between the development and evaluation data in healthcare setting, eligibility criteria, outcome,and predictors
**Ethical approval**	17	D;E	Name the institutional research board or ethics committee that approved the study and describe the participant informed consent or the ethics committee waiver of informed consent
**Open science**			
**Funding**	18a	D;E	Give the source of funding and the role of the funders for the present study
**Conflicts of interest**	18b	D;E	Declare any conflicts of interest and financial disclosures for all authors
**Protocol**	18c	D;E	Indicate where the study protocol can be accessed or state that a protocol was not prepared
**Registration**	18d	D;E	Provide registration information for the study, including register name and registration number, or state that the study was not registered
**Data sharing**	18e	D;E	Provide details of the availability of the study data
**Code sharing**	18f	D;E	Provide details of the availability of the analytical code§
**Patient and public involvement**			
**Patient and public involvement**	19	D;E	Provide details of any patient and public involvement during the design, conduct, reporting, interpretation or dissemination of the study or state no involvement
**Result**			
**Participants**	20a	D;E	Describe the flow of participants through the study, including the number of participants with and without the outcome and, if applicable, a summary of the follow‐up time. A diagram may be helpful
	20b	D;E	Report the characteristics overall and, where applicable, for each data source or setting, including the key dates, key predictors (including demographics), treatments received, sample size, number of outcome events, follow‐up time and amount of missing data. A table may be helpful. Report any differences across key demographic groups
	20c	E	For model evaluation, show a comparison with the development data of the distribution of important predictors (demographics, predictors and outcome)
**Model development**	21	D;E	Specify the number of participants and outcome events in each analysis (eg, for model development, hyperparameter tuning, model evaluation)
**Model specification**	22	D	Provide details of the full prediction model (eg, formula, code, object, application programming interface) to allow predictions in new individuals and to enable third‐party evaluation and implementation, including any restrictions to access or reuse (eg, freely available, proprietary)¶
**Model performance**	23a	D;E	Report model performance estimates with confidence intervals, including for any key subgroups (eg, sociodemographic). Consider plots to aid presentation
	23b	D;E	If examined, report results of any heterogeneity in model performance across clusters. See TRIPOD‐Cluster for additional details‡
**Model updating**	24	E	Report the results from any model updating, including the updated model and subsequent performance
**Discussion**			
**Interpretation**	25	D;E	Give an overall interpretation of the main results, including issues of fairness in the context of the objectives and previous studies
**Limitations**	26	D;E	Discuss any limitations of the study (such as a non‐representative sample, sample size, overfitting, missing data) and their effects on any biases, statistical uncertainty and generalizability
**Usability of the model in the context of current care**	27a	D	Describe how poor quality or unavailable input data (eg, predictor values) should be assessed and handled when implementing the prediction model
	27b	D	Specify whether users will be required to interact in the handling of the input data or use of the model, and what level of expertise is required of users
	27c	D;E	Discuss any next steps for future research, with a specific view to applicability and generalizability of the model

TRIPOD = Transparent Reporting of a multivariable prediction model for Individual Prognosis Or Diagnosis; AI = artificial intelligence.

* D = items relevant only to the development of a prediction model; E = items relating solely to the evaluation of a prediction model; D;E = items applicable to both the development and evaluation of a prediction model.

† Separately for all model‐building approaches.

‡ TRIPOD‐Cluster is a checklist of reporting recommendations for studies developing or validating models that explicitly account for clustering or explore heterogeneity in model performance (eg, at different hospitals or centres) 0.1920.

§ Relates to the analysis code, for example, any data cleaning, feature engineering, model building and evaluation.

¶ Relates to the code to implement the model to get estimates of risk for a new individual.

**TABLE A3:** Machine learning models included in the study with relevant method function in caret, type of model and modifiable tuning parameters.

Machine Learning Models			
Model	*method* Value in caret	Type	Tuning Parameters
Random Forest	ranger	Classification, Regression	mtry, splitrule, min.node.size
Neural Network	nnet	Classification, Regression	size, decay
Support Vector Machines with Linear Kernel	svmLinear	Classification, Regression	C
eXtreme Gradient Boosting	xgbLinear	Classification, Regression	nrounds, lambda, alpha, eta
Elasticnet	enet	Regression	fraction, lambda

**TABLE A4:** Overview of selected patient characteristics at baseline and percentage of missingness.

n = 963	Mean/Frequency/SD	Missingness, n (%)
Patient‐Related Preoperative Variables		
Sex, n (%)		0
Male	641 (68.5)	
Female	322 (31.5)	
Age [years], mean (SD)	63.90 (12.5)	0
BMI [[kg/m^2^], mean (SD)	28.0 (5.4)	40 (4.2)
Preoperative Hb [g/dl], mean (SD)	14.2 (1.8)	3 (0.3)
Preoperative creatinine [mg/dl], mean (SD)	1.1 (0.8)	7 (0.7)
Preoperative eGFR [ml/min], mean (SD)	72.4 (22.0)	5 (0.5)
Preoperative CKD stage, n (%)		5 (0.5)
1	252 (26.2)	
2	435 (45.2)	
3	174 (18.1)	
4	74 (7.7)	
5	12 (1.2)	
6	11 (1.1)	
Solitary kidney, n (%)	28 (2.9)	0
ASA classification, n (%)		251 (26.1)
1	121 (12.6)	
2	391 (40.6)	
3	194 (20.1)	
4	6 (0.6)	
MAP score, n (%)		121 (12.6)
0	126 (13.1)	
1	66 (6.9)	
2	115 (11.9)	
3	173 (18.0)	
4	261 (27.1)	
5	101 (10.5)	
**Tumour Related Variables**		
Multifocality, n (%)	39 (4.0)	0
Tumour size [cm], mean (SD)	4.4 (2.9)	33 (3.4)
RENAL score, mean (SD)	8.0 (2.0)	93 (9.7)
RENAL score, n (%)		93 (9.7)
Low: 4–6	213 (22.1)	
Intermediate: 7–9	419 (435)	
High: 10–12	239 (24.8)	
PADUA, mean (SD)	9.46 (2.12)	140 (14.5)
pT, n (%)		210 (21.8)
1a	380 (39.5)	
1b	184 (19.1)	
2a	51 (5.3)	
2b	12 (1.2)	
3a	115 (11.9)	
3b	6 (0.6)	
3c	2 (0.2)	
4	3 (0.3)	
pN, n (%)		216 (22.4)
0	731 (75.9)	
1	14 (1.5)	
X	2 (0.2)	
Grading, n (%)		272 (28.2)
1	250 (26.0)	
2	378 (39.3)	
3	46 (4.8)	
4	17 (1.8)	
R, n (%)		214 (22.2)
0	715 (74.4)	
1	33 (3.4)	
2	1 (0.1)	
Histology type, n (%)		3 (0.3)
benign	197 (20.5)	
Clear cell RCC	495 (51.4)	
Papillary RCC	166 (17.2)	
Chromophobe RCC	63 (6.5)	
Other RCC	31 (3.2)	
Malignant tumour, no RCC	8 (0.8)	
**Surgery Related Variables**		
Surgical technique, n (%)		0
Open partial nephrectomy	439 (45.6)	
Robot‐assisted partial nephrectomy	287 (29.8)	
Open radical nephrectomy	179 (18.6)	
Robot‐assisted radical nephrectomy	58 (6.0)	
Surgeon experience, n (%)		0
Inexperienced	143 (14.8)	
Experienced	820 (85.2)	
Surgery duration [min], mean (SD)	141.7 (47.7)	3 (0.3)
Estimated blood loss [ml], mean (SD)	356.5 (609.3)	172 (17.9)
Ischemia, n (%)	610 (63.3)	5 (0.5)
Ischemia time [min], mean (SD)	11.0 (9.9)	13 (1.3)
Drainage, n (%)	250 (26.0)	7 (0.7)
Intraoperative pleural opening, n (%)	276 (28.7)	0
Intraoperative opening of collective duct system, n (%)	341 (35.4)	3 (0.3)
Use of tissue adhesive sealant, n (%)	260 (27.0)	6 (0.6)
**Postoperative outcome variables**		
Clavien‐Dindo Classification, n (%)		0
0	661 (68.6)	
1	76 (7.9)	
2	140 (14.5)	
3a	53 (5.5)	
3b	20 (2.1)	
4a	8 (0.8)	
4b	2 (0.2)	
5	3 (0.3)	
Major complications [CDC ≥ 3], n (%)	86 (8.9)	0
AKI stage, n (%)		0
0	628 (66.1)	
1	249 (26.2)	
2	53 (5.6)	
3	20 (2.1)	
Acute kidney injury [AKI ≥ 1], n (%)	322 (33.4)	0
Duration of stay [days], mean (SD)	7.1 (3.8)	0

## References

[1] Pierorazio PM , Johnson MH , Patel HD , Sozio SM , Sharma R , Iyoha E , et al. Management of Renal Masses and Localized Renal Cancer: Systematic Review and Meta‐Analysis. J Urol. 2016;196(4):989–999. 10.1016/j.juro.2016.04.081 27157369 PMC5593254

[2] Simhan J , Smaldone MC , Tsai KJ , Canter DJ , Li T , Kutikov A , et al. Objective measures of renal mass anatomic complexity predict rates of major complications following partial nephrectomy. Eur Urol. 2011;60(4):724–730. 10.1016/j.eururo.2011.05.030 21621910 PMC3319121

[3] Reddy UD , Pillai R , Parker RA , Weston J , Burgess NA , Ho ETS , et al. Prediction of complications after partial nephrectomy by RENAL nephrometry score. Ann R Coll Surg Engl. 2014;96(6):475–479. 10.1308/003588414X13946184903522 25198982 PMC4474202

[4] Liu ZW , Olweny EO , Yin G , Faddegon S , Tan YK , Han WK , et al. Prediction of perioperative outcomes following minimally invasive partial nephrectomy: role of the R.E.N.A.L nephrometry score. World J Urol. 2013;31(5):1183–1189. 10.1007/s00345-012-0876-3 22544340

[5] Kowalewski KF , Egen L , Fischetti CE , Puliatti S , Juan GR , Taratkin M , et al. Artificial intelligence for renal cancer: From imaging to histology and beyond. Asian J Urol. 2022;9(3):243–252. 10.1016/j.ajur.2022.05.003 36035341 PMC9399557

[6] Garrow CR , Kowalewski KF , Li L , Wagner M , Schmidt MW , Engelhardt S , et al. Machine learning for surgical phase recognition: A systematic review. Ann Surg. 2021;273(4):684–693. 10.1097/SLA.0000000000004425 33201088

[7] Khene ZE , Bigot P , Doumerc N , Ouzaid I , Boissier R , Nouhaud FX , et al. Application of machine learning models to predict recurrence after surgical resection of nonmetastatic renal cell carcinoma. Eur urol oncol. 2023;6(3):323–330. 35987730 10.1016/j.euo.2022.07.007

[8] Suarez‐Ibarrola R , Hein S , Reis G , Gratzke C , Miernik A . Current and future applications of machine and deep learning in urology: a review of the literature on urolithiasis, renal cell carcinoma, and bladder and prostate cancer. World J Urol. 2020;38(10):2329–2347. 10.1007/s00345-019-03000-5 31691082

[9] Doyle PW , Kavoussi NL . Machine learning applications to enhance patient specific care for urologic surgery. World J Urol. 2022;40(3):679–686. 10.1007/s00345-021-03738-x 34047826

[10] Collins GS , Reitsma JB , Altman DG , Moons KGM . Transparent reporting of a multivariable prediction model for individual prognosis or diagnosis (TRIPOD): The TRIPOD statement. BMJ. 2015;350:g7594. 10.1136/bmj.g7594 25569120

[11] Collins GS , Moons KGM , Dhiman P , Riley RD , Beam AL , Van Calster B , et al. TRIPOD+AI statement: Updated guidance for reporting clinical prediction models that use regression or machine learning methods. BMJ. 2024;385:e078378. 10.1136/bmj-2023-078378 38626948 PMC11019967

[12] Dindo D , Demartines N , Clavien PA . Classification of surgical complications: A new proposal with evaluation in a cohort of 6336 patients and results of a survey. Ann Surg. 2004;240(2):205–213. 10.1097/01.sla.0000133083.54934.ae 15273542 PMC1360123

[13] Thomas ME , Blaine C , Dawnay A , Devonald MAJ , Ftouh S , Laing C , et al. The definition of acute kidney injury and its use in practice. Kidney Int. 2015;87(1):62–73. 10.1038/ki.2014.328 25317932

[14] Jung JO , Crnovrsanin N , Wirsik NM , Nienhüser H , Peters L , Popp F , et al. Machine learning for optimized individual survival prediction in resectable upper gastrointestinal cancer. J Cancer Res Clin Oncol. 2023;149(5):1691–1702. 10.1007/s00432-022-04063-5 35616729 PMC10097798

[15] Jung JO , Pisula JI , Bozek K , Popp F , Fuchs HF , Schröder W , et al. Prediction of postoperative complications after oesophagectomy using machine‐learning methods. Br J Surg. 2023;110(10):1361–1366. 10.1093/bjs/znad181 37343072

[16] Van Poppel H , Da Pozzo L , Albrecht W , Matveev V , Bono A , Borkowski A , et al. A prospective randomized EORTC intergroup phase 3 study comparing the complications of elective nephron‐sparing surgery and radical nephrectomy for low‐stage renal cell carcinoma. Eur Urol. 2007;51(6):1606–1615. 10.1016/j.eururo.2006.11.013 17140723

[17] Liu X , Faes L , Kale AU , Wagner SK , Fu DJ , Bruynseels A , et al. A comparison of deep learning performance against health‐care professionals in detecting diseases from medical imaging: a systematic review and meta‐analysis. Lancet Digit Health. 2019;1(6):e271–e297. 10.1016/S2589-7500(19)30123-2 33323251

[18] Charilaou P , Battat R . Machine learning models and over‐fitting considerations. World J Gastroenterol. 2022;28(5):605–607. 10.3748/wjg.v28.i5.605 35316964 PMC8905023

[19] Lanera C , Berchialla P , Sharma A , Minto C , Gregori D , Baldi I . Screening PubMed abstracts: is class imbalance always a challenge to machine learning? Syst Rev. 2019;8(1):317. 10.1186/s13643-019-1245-8 31810495 PMC6896747

[20] Vickers AJ , van Calster B , Steyerberg EW . A simple, step‐by‐step guide to interpreting decision curve analysis. Diagn Progn Res. 2019;3(1):18. 10.1186/s41512-019-0064-7 31592444 PMC6777022

